# P-1118. A Summer COVID-19 Outbreak at a Community Living Center within a Rural Veterans Affairs Hospital in the Midwest

**DOI:** 10.1093/ofid/ofaf695.1313

**Published:** 2026-01-11

**Authors:** Kelly Biermann, Melissa Moore, Karen Quella, Aurora E Pop-Vicas

**Affiliations:** University of Wisconsin School of Medicine and Public Health, Madison, WI; Tomah VAMC, Tomah, Wisconsin; Tomah VA Medical Center, Tomah, Wisconsin; University of Wisconsin School of Medcine and Public Health, Madison, WI

## Abstract

**Background:**

Community living centers (CLC) are Veterans Affairs (VA) nursing homes that provide a variety of medical services, such as short- and long-term rehabilitation, behavioral and psychiatric care, and end-of-life care. They emphasize social and recreational activities and strive to create a home-like environment. There is little known about the impact of post-pandemic nosocomial COVID-19 outbreaks in this health-care setting.

**Figure:**
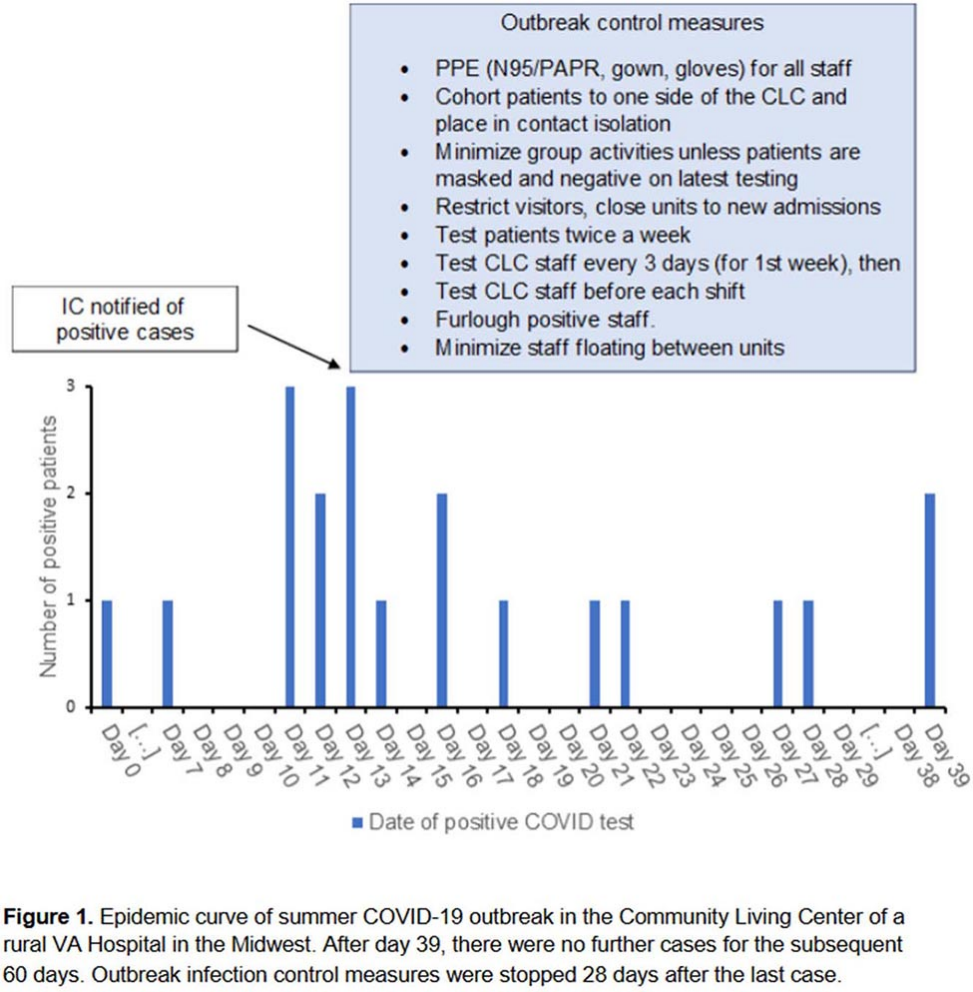

**Methods:**

We describe a SARS-CoV-2 outbreak in a rural CLC in the Midwest during June 27, 2024-August 5, 2024, at a time when the COVID-19 endemic transmission was high in the surrounding community. Initial cases were identified by PCR testing of veterans with upper respiratory virus symptoms, followed by liberal testing for asymptomatic carriage for both veterans and staff. Whole genome sequencing was performed on all available clinical isolates to identify transmission clusters.

**Results:**

A total of 21 veterans (22% of CLC population) were diagnosed with COVID-19 on 6 different CLC units (3 within adjacent buildings connected by tramway, 3 on different standalone buildings) with shared staff. Among the positive cases, 6 veterans (29%) were asymptomatic. Four veterans required oxygen and were transferred to an acute care unit for treatment with remdesivir and dexamethasone, and 5 veterans received treatment with Paxlovid in the CLC. All veterans recovered to their baseline health within 30 days, except for one hospice veteran who died. A total of 34 staff members who worked in the affected units were positive during the outbreak. Ten isolates (4 veterans, 6 staff) were genetically sequenced identifying two SARS-CoV-2 strains (LB 1.3.2. and JN1.16.1) among both veterans and staff. Figure 1 shows the epidemic curve for the veterans affected and the infection control measures taken to control the outbreak.

**Conclusion:**

The infection control measures employed to control this outbreak were resource-intensive, costly, and disruptive, but necessary to stop further spread. Future studies should determine what procedures should be instituted in this unique healthcare setting to prevent further outbreaks during seasons of high respiratory virus circulation in the community.

**Disclosures:**

All Authors: No reported disclosures

